# Base Catalytic Approach: A Promising Technique for the Activation of Biochar for Equilibrium Sorption Studies of Copper, Cu(II) Ions in Single Solute System

**DOI:** 10.3390/ma7042815

**Published:** 2014-04-09

**Authors:** Sharifah Bee Abdul Hamid, Zaira Zaman Chowdhury, Sharifuddin Mohammad Zain

**Affiliations:** 1 Nanoscience and Catalysis Center (NANOCAT), University Malaya, Kuala Lumpur 50603, Malaysia; E-Mail: sharifahbee@um.edu.my; 2 Department of Chemistry, Faculty of Science, University Malaya, Kuala Lumpur 50603, Malaysia; E-Mail: smzain@um.edu.my

**Keywords:** adsorption, kinetics, isotherm, heavy metals, copper, Cu(II), single solute system

## Abstract

This study examines the feasibility of catalytically pretreated biochar derived from the dried exocarp or fruit peel of mangostene with Group I alkali metal hydroxide (KOH). The pretreated char was activated in the presence of carbon dioxide gas flow at high temperature to upgrade its physiochemical properties for the removal of copper, Cu(II) cations in single solute system. The effect of three independent variables, including temperature, agitation time and concentration, on sorption performance were carried out. Reaction kinetics parameters were determined by using linear regression analysis of the pseudo first, pseudo second, Elovich and intra-particle diffusion models. The regression co-efficient, R^2^ values were best for the pseudo second order kinetic model for all the concentration ranges under investigation. This implied that Cu(II) cations were adsorbed mainly by chemical interactions with the surface active sites of the activated biochar. Langmuir, Freundlich and Temkin isotherm models were used to interpret the equilibrium data at different temperature. Thermodynamic studies revealed that the sorption process was spontaneous and endothermic. The surface area of the activated sample was 367.10 m^2^/g, whereas before base activation, it was only 1.22 m^2^/g. The results elucidated that the base pretreatment was efficient enough to yield porous carbon with an enlarged surface area, which can successfully eliminate Cu(II) cations from waste water.

## 1. Introduction

The impending inadequacy of fossil fuels has necessitated the search for alternative sources to produce advanced carbonaceous materials. In view of that, biomass is considered as one of the most promising sources of carbon, as well as of renewable chemical feedstock. The challenge lies in depolymerizing the complex lignocellulosic matrix to yield carbon with desirable properties for a specific application. Biomass resources are renewable, readily available and would not contribute to the overall degradation of the environment; in a word, sustainable. Thus, biomass can be considered as a greener alternative for fossil fuels, which is produced by living things [1,2]. Thermochemical transformation of biomass can yield value-added products, replace fossil fuels with little or zero emission of toxic chemicals, while closing the loop of the carbon cycle [2,3]. A diversity of feedstock, including residual stalks, leaves, straw, roots, husk, nut shells, wood chips, saw dust and animal husbandry waste, constitutes biomass. Although there is a growing trend for utilizing biomass substrate to yield useful chemicals, this potential resource is largely under-utilized or even left to decompose and openly burnt, especially in developing countries, which do not have strong regulations to prevent such pollution practices. Direct combustion of agricultural residues leads to the addition of greenhouse gasses and, thus, contributes to air pollution to a great extent. In this context, these residues can be chemically treated before pyrolysis to yield biochar with effective surface characteristics for further applications. To date, a lot of initiatives has been taken to prepare carbonaceous adsorbent from biomass substrate, which can preferentially find its application in waste water treatment [4–9].

The presence of heavy metals in aqueous stream is alarming, and presently, it is considered one of the most crucial environmental problems [10,11]. This affects not only aquatic life and human beings, but also the overall ecosystem negatively. Some industrial activities together with anthropogenic activities have enriched water bodies with heavy metals [12]. The metallic cations are non-biodegradable and usually present in very low concentrations in the aqueous stream [11–15]. Copper (Cu) is considered one of the hazardous pollutants for aqueous effluents, due to its high toxicological effects. Divalent cations of copper, Cu(II), frequently enter into the process stream by mining, brass manufacturing, smelting and electroplating industries. They can cause lung cancer and gastrointestinal disorder in human. Recently, the preparation of low-cost adsorbent derived from agricultural residues has gained increasing interest by researchers. Conversion of biomass residues for carbon adsorbent preparation has those advantages of a reduced amount of sludge production, biodegradability and the simplicity of design. Pyrolysis of agricultural wastes, such as corn cob [16], apricot stone [17,18], date pits [19], peach stone [20], corn stover [21], coconut shell [22], grape seed [23], cherry stone [24], rice husk [25], *etc*., has been done recently to yield activated char. A wide variety of products, including char, gases, light oils and tars, can be procured from the pyrolysis of biomass. However, the percentage yield of chemicals during pyrolysis processes can be significantly changed by using catalysts or with chemical pretreatment [26]. During biomass pyrolysis, the organic content burns to produce tar, which precipitates onto the surface of the carbon and blocks the pores. Metallic content present in ash residues of biomass substrate partially reduces the formation of tars, which is undesirable for the synthesis of porous carbon [26,27]. The presence of acidic or basic types of catalyst promotes the formation of carbon with an extended surface area, along with significant porosities. The presence of inorganic compound in low amounts initiates dehydration reactions and char yield through the pyrolysis of tars [26,27]. It is observed that alkaline metal hydroxide, especially potassium hydroxide (KOH), can be used to produce activated carbon from coal and char [28]. The efficiency of activated carbon as an ideal adsorbent material is attributed to its porous texture, as well as surface functional groups. It has been anticipated that by controlling the process parameters during oxidation or activation, the presence of certain inorganic and organic groups on the surface of activated carbon particles can be enhanced [28].

The objective of this study is to observe the role of potassium hydroxide (KOH) for the pyrolysis of biochar synthesized from the dried peel or exocarp of purple mangostene fruits. The study investigates the feasibility of using the activated biochar for the elimination of Cu(II) cations in single solute system. The effects of process parameters, such as initial concentration, contact time and temperature, were discussed for a single solute system. The physiochemical parameters, including equilibrium isotherm, kinetics and thermodynamics studies with the necessary surface characteristics of the prepared char samples, are presented in a subsequent section. The future perspective of this work is to apply the prepared biochar for a multi-solute system and column mode studies for lab-scale, as well as large-scale, pilot plant experiments, which consequently can results in the commercialization of the end products.

## 2. Results and Discussion

The char used here for adsorption studies was prepared in two steps by using the physiochemical activation method. At the first stage, the sample was prepared at low temperature, which was unactivated. The physiochemical characteristics of the unactivated char demonstrated that further activation was needed to enhance the surface area, as well as the porosity of the sample to ensure maximum removal of Cu(II) cations from the waste stream.

### 2.1. Physiochemical Properties of Unactivated and Activated Biochar

Surface functional groups containing oxygen play a vital role in the adsorption process. This will initiate chemical bonding between adsorbent and adsorbate species. The major peaks for the chemical groups that can efficiently contribute to the removal of Cu(II) cations are listed in Table 1. A broad peak between 3321.66 and 3756.44 cm*^−^*^1^ was observed from the FTIR spectra of the raw, unactivated and activated biochar samples, which can be ascribed to the –OH stretch of hydroxyl functional group. The peak observed around 1756.77 cm^−1^ in the raw biomass disappeared after carbonization. The trend of the FTIR spectrum for the lignocellulosic precursors, as well as carbonized and activated samples contains some main peaks, which are almost similar. Some major peaks around 2800–2900 cm^−1^, 1500–1610 cm^−1^ and 1100 cm^−1^ are related to the C–H stretching of alkane, the C=C stretching of the aromatics and the C–O–C stretching vibration of the esters, ether and phenol groups. The weak to medium peaks located at 502.50–998.50 cm^−1^ are assigned to the C–H out-of-plane bending, as well as the O–H stretching vibrations of the C–O–H band. The weak peak detected around 1400–1550 cm^−1^ on the spectrum of the prepared char is due to the C=O stretching vibration of carboxylate anions. After impregnation and activation by using KOH, a lot of peaks shifted their frequency level or, in some cases, disappeared. A similar phenomenon has been observed by previous researchers. Yang and Lua (2003) [29] reported that different oxygen groups, which were present in the raw pistachio nut shell, disappeared after the heat treatment, causing the aromatization of the carbon structure. It was reported, also, that after carbonization, the chemical structure of the raw date pit was changed significantly. Some aliphatic C–H groups were lost, whereas some aromatic C=C and oxygen groups were developed after the activation process [30]. When the activation temperature was increased, some poly aromatic structures were formed, due to the destruction of the C=O and C–O groups. After KOH treatment of rice straw, the peak intensities of the ester groups and phenolic ether groups were decreased significantly. The researchers described that pyrolysis in the presence of KOH had destroyed the lignin structure containing the ester and ether linkages after the activation of rice straw [31].

It was earlier reported in the literature that the adsorption of heavy metals is strongly dependent on the pH value of the medium. At a pH around 2–3, H^+^ and H_3_O^+^ ions compete with positive metallic cations, resulting in lower removal efficiency [32,33]. At pH 6, copper can exist either in metallic Cu(II) cations or in its hydroxide form in water. The –OH group present on the surface of the prepared sample can undergo the following reactions to facilitate the overall removal process.

1. (−RO^+^H_2_) + Cu^2+^ → (RO) Cu + 2H^+^
2. (−RO^+^H_2_) + Cu (OH)^+^ →(−RO) CuOH + 2H^+^
3. nR−COOH + Cu^2+^ → (R−COO)_n_ Cu + nH^+^

The prepared sample contains carboxylate (–COOH) groups. –COOH groups present on the surface of the activated carbon can dissociate around pH 5, as they have pKa values of three to five. Cu(II) can form surface complexes according to reaction Scheme 3, due to the presence of carboxylate anions onto the activated sample. Here, R represents the surface of the activated biochar sample. A similar reaction scheme for the adsorptive removal of Cu(II) was reported by using orange peel, saw dust and bagasse [34]. However, at basic pH, Cu(II) cations might precipitate [35]. That is why, to avoid cumulative effect of adsorption and precipitation, adsorption studies were conducted at pH 5.5.

**Figure 1.**
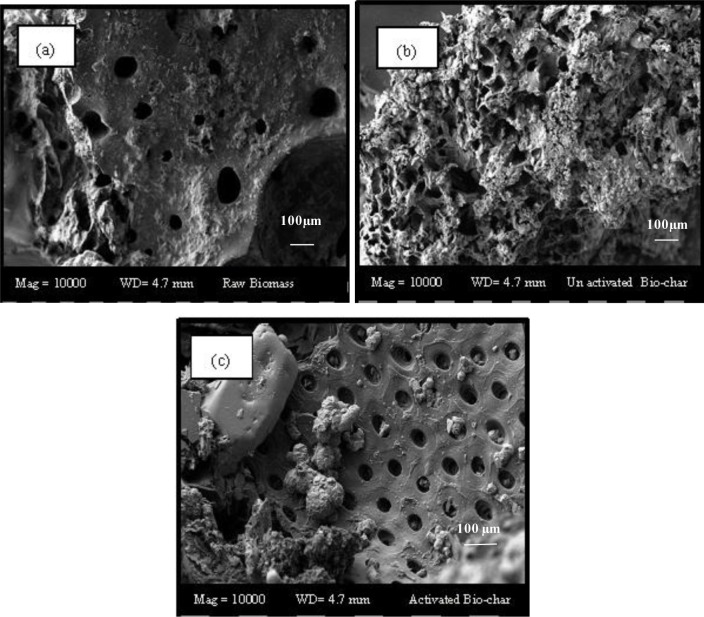
SEM images (10,000×) of (**a**) raw biomass; (**b**) unactivated and (**c**) activated char.

Figure 1a–c shows the SEM micrograph of raw biomass, unactivated and activated biochar. As can be observed from Figure 1a, the surface textures of the raw biomass were comparatively rough and uneven, with some minor pores observed on the surface. The unactivated char contained few pores, with some lumps of tarry deposits, as shown by Figure 1b. After the activation process, a significant amount of pores were developed on the surfaces of the biochars, which were circular in shape. This implies that KOH has successfully catalyzed the formation of new pores in the presence of CO_2_ gas flow at the temperature and time applied for the process. A similar trend has been observed for producing activated carbon from guava seed [36]. It was observed that one-step carbonization alone failed to create sufficient porosities, due to the incomplete decomposition of organic constituents present in the carbonaceous precursors. In that case, the pores were significantly blocked by the residues of carbonization products, leading to a reduced surface area with less porosity [36]. The surface area along with the micropore volume has been summarized in Table 2. The activation process in the presence of KOH showed the drastic increment of the surface area with an adequate micropore volume in the activated sample.

The ultimate analysis of the samples showed that the carbon content increased and the hydrogen content decreased significantly after the activation of the biochar, illustrating that the activation technique applied here is efficient enough to develop activated biochar suitable for the uptake of divalent cations from the waste stream. This is because at a high temperature, the organic substances present inside the carbon matrix become unstable. Their bonding is broken, and volatile substances are discharged both as gas and liquid products.

### 2.2. Effect of Concentration and Agitation Time

**Figure 2.**
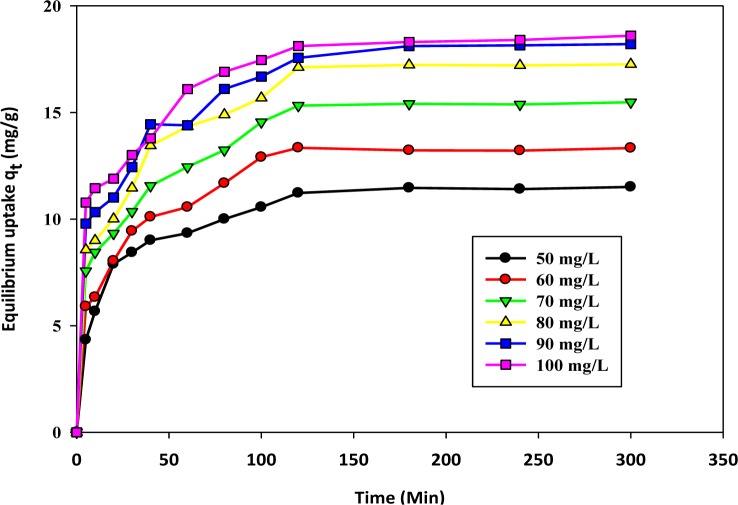
The effect of concentration with contact time and equilibrium uptake.

The effect of the concentration and agitation time has been illustrated by Figure 2. The agitation time influences the formation of the external film, which creates a boundary layer over the surface of the sorbent. The linear plots clearly indicate that for all the concentration ranges, the amount of adsorbed cation, *q_t_* (mg/g), increased with the lapse of time. However, there is a saturation limit after which the system has reached a state of dynamic equilibrium; beyond that, no significant amount of cations are eliminated from the medium. This maximum level is known as the “equilibrium level”. At this equilibrium point, the cations desorbing from the activated biochar are almost the same as the amount of cations adsorbed onto the surface of the prepared biochar. After this stage, the curve becomes parallel to the x-axis, which means a negligible amount of sorption. The curve obtained here shows two distinct regions of sorption. The initial region is steep, fast and achieved quickly. The second stage is relatively slow near the equilibrium. At the preliminary stage of adsorption, significant numbers of active surface sites are available, which can readily combine with the cations. After a certain period of time, the active surface sites have been exhausted. Thus, it was difficult for them to be occupied by the remaining cations. This difficulties arises due to the repulsive forces between the solute (cations attached onto the solid surface) and the free ions (cations dispersed in the liquid phases) remaining in the solution. The adsorption uptake at equilibrium is found to increase with increasing initial cation concentrations. This is because at a higher initial concentration, the driving force for mass transfer becomes larger. Consequently, there will be more equilibrium uptake. This phenomenon was reported earlier for adsorption studies of divalent cations of Pb(II), Cd(II) and Ni(II) onto sawdust [37].

### 2.3. Evaluation of Kinetics Parameters

The evaluation of the kinetics parameters is important, as these can give insight into the rate and type of sorption process. The correlation coefficient, *R*^2^, and the standard deviation, ∆*q* (%), have been determined in several other works in the literature to validate the applicability of specific types of kinetic equations. The equation can be written as:

$$\Delta q(\%)=100\sqrt{{\frac{\sum \left[\left(q_{t,\exp}-q_{t,\mathrm{cal}}/q_{t,\exp}\right)\right]}{\left(N-1\right)}}^{2}}$$ (1)

where *N* means the number of data points and and (mg/g) are the experimental and calculated adsorption uptake at time *t*, respectively [38,39].

The pseudo first order kinetic model has been applied extensively to analyze sorption data by using the following Equations (1) and (2) [40,41]:

$$\log(q_{e}-q_{t})=\log\;q_{e}-\frac{K}{2.303}t$$ (2)

$$h=K_{1}q_{e,\mathrm{cal}}$$ (3)

**Figure 3.**
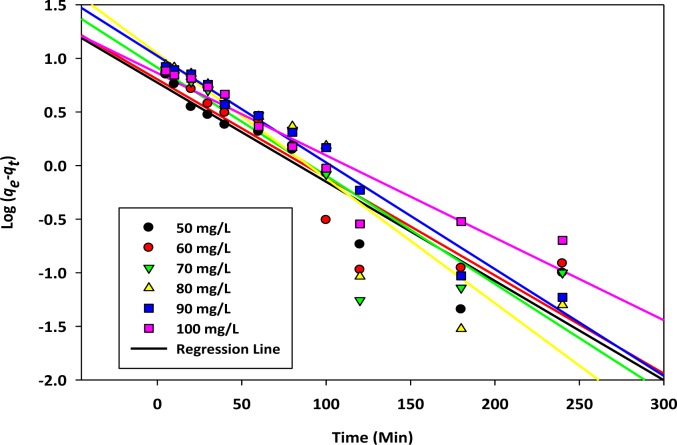
Pseudo first order kinetics of copper, Cu(II) cation sorption onto activated biochar.

$\frac{dq}{dt}=K_{1}(q_{e}-q_{t})$. Here, *K*_1_ (L/min) represents the first order rate constant; *h* (mg/g·min^−1^) represents the initial rate of sorption; *q_e_* (mg/g) and *q_t_* (mg/g) are the uptake of the targeted cation at the equilibrium contact time and at any time, *t* (min), respectively. Linear regression analysis of ln (*q_e_*−*q_t_*) *versus t* (min) were carried out by using Sigma Plot 10 and shown in Figure 3.

**Figure 4.**
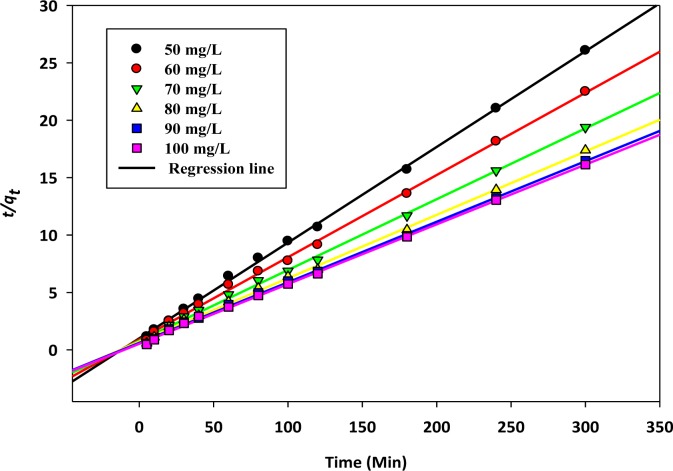
Pseudo second order kinetics of copper, Cu(II) cations sorption onto activated biochar.

Equilibrium kinetics data were analyzed by using the linear form of the pseudo second order equation and are expressed by the following Equations (4) and (5) [12,42,43].

$$\frac{t}{q_{t}}=\frac{1}{K_{2}q_{e}^{2}}+\frac{1}{q_{e}}t$$ (4)

$$h=K_{2}q_{e,cal}^{2}$$ (5)

where the rate constant of second order adsorption is *K*_2_ (g/mg·min^−1^) and *h* (g/mg·min^−1^) is the initial rate of sorption. The linear regression analysis of *t/qt*, *versus t* (min) was conducted and is illustrated in Figure 4. The calculated parameters for first and second order kinetics parameters are evaluated and listed in Table 3.

**Figure 5.**
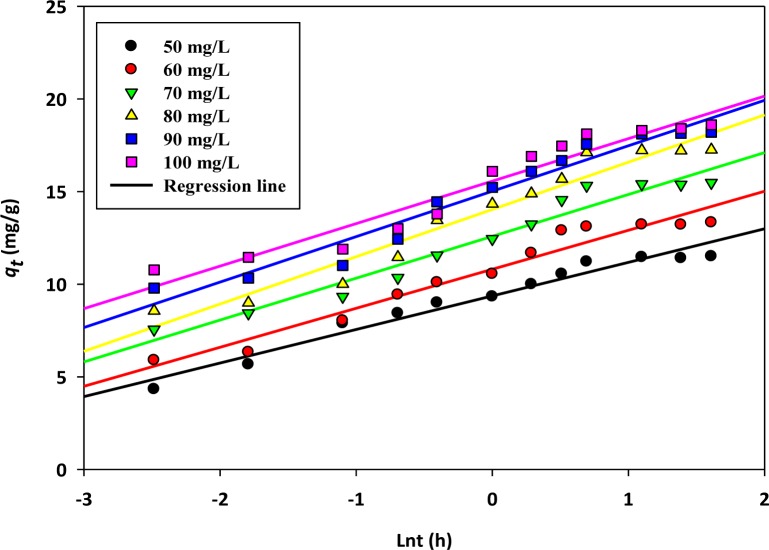
Elovich kinetics of copper, Cu(II) cations sorption onto activated biochar.

The equilibrium data were further analyzed to elucidate the chemisorption studies by using the following equation [39]:

$$q_{t}=\frac{1}{b}\ln(ab)+\frac{1}{b}\ln t$$ (6)

where a (mg/g·h^−1^) represents the rate of sorption at the initial stage and *b* (mg/g) represents the energy for activation. The linear plots of the Elovich model are illustrated in Figure 5, and the estimated model constants are listed in Table 4.

From Tables 3 and 4, it is observed that the *R*^2^ values obtained for the pseudo second order and Elovich equations are better compared to the pseudo first order model. Furthermore, the values of ∆*q*% obtained for the Elovich and second order kinetics are smaller than the first order kinetics. This confirms the involvement of chemisorption in the rate controlling step. It is observed that the values of 1/*b* ln (*ab*) increase with the increase of the initial concentration range studied. This trend is expected, because as the concentration range increases, a relatively large number of adsorbate ions will effectively collide with the active sites of the adsorbents to form surface complexes. Eventually, more uptakes by the prepared adsorbents will be observed.

### 2.4. Reaction Mechanism: Intra-Particle Diffusion

The intra-particle diffusion model has been applied by several researchers to predict the role of diffusion, and it is depicted by the following equation [44]:

$$q_{t}=k_{dif}t^{0.5}+C_{i}$$ (7)

**Figure 6.**
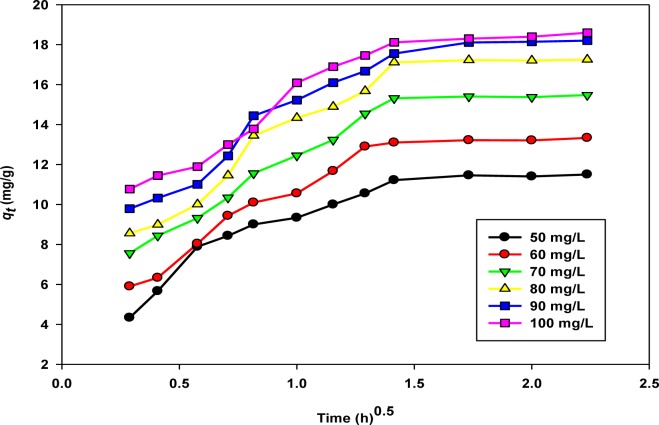
Intra-particle diffusion of copper, Cu(II) cations sorption onto activated biochar.

The intra-particle diffusion plots of *q_t_* (mg/g) *versus t*^0.5^ (h) are shown by Figure 6.

From the figure, almost two distinct regions of adsorption were observed. The first, steeper region reflects a rapid rate of sorption or external surface sorption. The second region of the curve represents the slower adsorption stage. The straight lines did not pass through the origin, which implies that along with the pore diffusion, various alternative mechanisms are involved in the rate controlling stage. The rate constants are listed in Table 5.

### 2.5. Evaluation of Isotherm Parameters in a Single Solute System

To interpret the isotherm parameters, three types of model equations, namely Langmuir, Freundlich, and Temkin, were used. Maximum monolayer adsorption capacity, *q*_m_ (mg/g) and K_L_, the Langmuir adsorption constant (L/mg) representing the binding energy for sorption under predefined reaction conditions, were measured from the Langmuir model expressed by the following equation [45]:

$$q_{e}=\frac{K_{\text{L}}C_{e}}{1+a_{L}C_{e}}$$ (8)

Linearization of Equation (8) will give Equation (9):

$$\frac{C_{e}}{q_{e}}=\frac{1}{q_{\max}K_{\text{L}}}+\frac{1}{q_{\max}}C_{e}$$ (9)

Another constant, known as the dimensionless factor, *R*_L_, of the Langmuir model is given by:

$$R_{\text{L}}=\frac{1}{1+K_{\text{L}}C_{o}}$$ (10)

In this present investigation, *R*_L_ values are calculated for the highest initial concentration studied here (100 mg/L). The values of separation factor *R*_L_ can give a precise idea about the types of isotherm, as summarized in Table 6.

The Freundlich isotherm is used to show the surface heterogeneity, which shows the multilayer adsorption properties of the adsorbent. The empirical equation is based on the hypothesis that the reactive sites over the adsorbate are distributed exponentially with the heat of the sorption process [46]. This is represented by:

$$q_{e}=K_{f}C_{e}{}^{1/n}$$ (11)

The above equation can be linearized to analyze the data and is represented by:

$$\ln q_{e}=\ln K_{f}+\frac{1}{n}\ln C_{e}$$ (12)

Here, *K*_f_ (mg/g) is the affinity factor of the cations towards the adsorbent and 1/*n* represents the intensity of the adsorption [46].

The Temkin isotherm presumes that the heat involved in the adsorption of the adsorbate in a layer would gradually decline linearly with the extent of surface exposure, due to the adsorbent-adsorbate interactions. Equilibrium data have been further analyzed by the Temkin isotherm and are expressed by [47]:

$$q_{e}=\frac{RT}{b}\ln K_{T}\ln C_{e}$$ (13)

Equation (13) can be linearized as:

$$q_{e}=\frac{RT}{b}\ln K_{T}+\frac{RT}{b}\ln C_{e}$$ (14)

Here, *RT*/*b* = B (J/mol) denotes the Temkin constant; which depicts the heat of the sorption process; whereas *K*_T_ (L/g) reflects the equilibrium binding constant; R (8.314 J/mol·K) is the universal gas constant; and *T*° (K) is the absolute solution temperature [47].

In this work, relatively better linearity for the Langmuir model was observed with correlation coefficients of 0.984 to 0.997. The overall trend obtained for the Langmuir separation factor, *R*_L_, and the Freundlich exponent, 1/*n*, being below one for all the temperature ranges studied, represent favorable adsorption processes. A similar trend for *R*_L_ and 1/*n* were observed for Mn(II) sorption onto raw and acid treated corncob biomass [48]. This showed good linearity for the Langmuir and Freundlich model, where the exponent, 1/*n*, below a value of one represented the normal Langmuir isotherm [48].

### 2.6. Evaluation of Thermodynamics Parameters in a Single Solute System

The thermodynamic characterization of the sample gave the values of the Gibbs free energy (*∆G°*), enthalpy (*∆H°*) and entropy (*∆S°*) of the sorption process [32]. The following linear equations were used to interpret the equilibrium data:

$$\ln\;K_{L}=\frac{S}{R}-\frac{H}{RT}$$ (15)

$$G=RT\ln K_{L}$$ (16)

Here, constant *K*_L_ (L/mg) was determined earlier by using a linear form of the Langmuir equation at three different temperatures. R is the universal gas constant (8.314 J/mol·K); and *T* is the absolute temperature in the Kelvin scale. The thermodynamic parameters were calculated from linear plots of ln *K*_L_ *versus* 1/*T* and are listed in Table 8. The enthalpy (*∆H°*) calculated here showed a positive magnitude. This implies that the process was endothermic [38,39]. This observation was further supported by the Langmuir maximum monolayer capacity, *q*_m_; and the Freundlich affinity factor, *K*_F_, tabulated earlier in Table 7. That is why the successive increase of temperature from 30 to 70°C had increased the values of the aforementioned parameters. Thus, it can be concluded that the removal percentages of Cu(II) cations were favored by a high temperature. Increasing the temperature increases the velocity of adsorbate species towards the interior of the adsorbent. The positive value of entropy, (*∆S°*), reflected the increased degree of freedom with some changes inside the structure of the activated biochar during the adsorption process. Similar phenomena had been depicted earlier for the elimination of divalent cations of lead from synthetic water applying palm ash residues activated by NaOH [32]. The Gibbs free energy changes, (*∆G°*), evaluated for all the temperatures under investigation were negative. This indicated that the sorption process was feasible and spontaneous.

## 3. Experimental

### 3.1. Preparation of Activated Biochar

After the extraction of the juicy endocarp, the waste peel or exocarp of mangostene fruits are not utilized properly, decompose or sometimes are burned to produce smoke by agro-industries. The dried peels were collected and cut into a 2–4 mm size. The samples were washed with hot deionized water and dried at 105°C for 24 h. The final char samples were obtained by the activation of dried peel by the two-stage activation method. Initially, 50 g of dried biomass samples were carbonized in the presence of purified nitrogen gas at 400°C for 2 h. The unactivated biochar thus obtained was stored in air-tight containers and sent for preliminary characterization studies. It was further impregnated with a base catalyst of KOH. During impregnation, the char and KOH ratio was kept at 1:1. Five-hundred millimeters of water were added with the char and KOH pellets and heated at 90–100°C for 6 h to initiate the disruption of residual lignocellulosic linkages with subsequent removal of unburnt tarry substances from the char. The mixture was kept overnight in an oven at 105°C to dry the sample completely. The unclogging of pores to enhance the surface area of the final product was further facilitated by a secondary stage of pyrolysis in the presence of carbon dioxide gas flow at a temperature of 700°C for 2 h. The activated biochar obtained in this process was washed vigorously to obtain a sample free from unreacted alkali. The final samples obtained were dried and stored in an air-tight container for further studies.

### 3.2. Preparation of Single Solute System

The single solute system of Cu(II) ions having a concentration of 1000 mg/L was prepared by dissolving the required amount of CuCl_2_·2H_2_O salt. The batch adsorption test was carried out by varying the concentration from 50 to 100 mg/L, where the concentration gap was kept at 10 mg/L for each sample. The stock solution was diluted to have the required concentration.

### 3.3. Physiochemical Properties of Unactivated and Activated Biochar

Fourier transform infrared (FTIR) spectra of the prepared samples were measured from 400 to 4000 cm^−1^ (Model Perkin Elmer FTIR-2000, Perkin Elmer, New York, NY, USA). FESEM images were taken to study the surface morphologies of raw biomass, unactivated and activated biochar. Elemental analysis of the prepared samples in terms of carbon, hydrogen, nitrogen and others was performed by using the Elemental Analyzer (PerkinElmer-2400, Tokyo, Japan).The surface area along with micropore volume and the area of the prepared biochar were analyzed by the Autosorb-1, Quantachrome Autosorb surface analyzer (Nova, FL, USA). Before performing the nitrogen gas adsorption-desorption at 77 K, the prepared char samples were outgassed under vacuum at 300°C for 4 h to remove any moisture content from the solid surface. At a constant temperature, a relative pressure of nitrogen gas flow over the sample inside the closed vessel of the surface area analyzer will be varied, and the volume of gas adsorbed will be recorded. The volume of gas adsorbed at different pressures was plotted until the equilibrium contact time. The porous texture of the prepared samples were obtained by using the BET equation to N_2_ adsorption at 77 K. The abovementioned procedure was automatically performed by software (Micropore version 2.26, Nova, FL, USA) available within the instrument.

### 3.4. Batch Adsorption Studies

Approximately 0.2 gm of the prepared char samples were placed with 50 mL solutions of the desired concentration range of Cu(II) ions. The equilibrium data were taken at pH 5.5 for the temperatures of 30°C, 50°C and 70°C for isotherm modeling and thermodynamic characterization of the sample. The solution containing activated char samples were agitated at 150 rpm. After a predetermined time interval, the residual metal ion concentration of the cations was analyzed. The loading of Cu(II) ions onto the active sites of the char was calculated by the following equation [32]:

$$q_{e}=\frac{(C_{0}-C_{e})V}{W}$$ (17)

Here, *q_e_* (mg/g) represents the amount of ion adsorbed at equilibrium; *C*_0_ is the initial concentration of the adsorbate; *C_e_* (mg/L) is the liquid-phase concentrations of Mn(II) ions at equilibrium conditions; *V* (L) is the volume of the synthetic solution; and *W* (g) is the mass of the biochar used [32,49].

## 4. Conclusions

In this study, biochar has been activated by using KOH to eliminate Cu(II) ions from synthetic solutions. After the preliminary stage of pyrolysis, the prepared char obtained has less surface area with an inadequate pore size distribution. In view of that, KOH was used as a base catalyst for further activation of the sample during the secondary pyrolysis stage in the presence of carbon dioxide. The extensive enlargement of the surface area after the base catalyzed pyrolysis process reflected that the physiochemical activation technique applied here was efficient enough to produce mesopore structured carbon. According to the experimental data, the activated sample showed a higher surface area than the untreated one. Equilibrium data were fitted to four types of kinetic models (pseudo first order, pseudo second order, Elovich and intra-particle diffusion), where the best correlation was obtained for the pseudo second order kinetics. Isotherm data were fitted to the Langmuir, Freundlich and Temkin isotherm models. A higher correlation coefficient was obtained for the Langmuir isotherm. Increasing temperature had a positive impact on the removal efficiency, implying the endothermic nature of sorption. The findings indicate that the activated char can be used to a great extent for treating Cu(II) contaminated effluents.

## Figures and Tables

**Table 1.** Analysis of the Fourier transform infrared (FTIR) spectra of the raw biomass, unactivated and activated biochar.

Number	Peak Frequency			Assignment
	Raw Biomass	Unactivated Biomass	Activated Biomass	
1	–	502.50	–	C–H bending
2	603.77, 610.74	602.70	–	C–O–H bending
3	788.77	762.70	768.66	C–H out-of-plane bending of benzene derivatives
4	805.77	801.70	804.77	C–H out-of-plane bending of benzene derivatives
5	–	998.50	–	O–H bending
6	1110.77, 1189.77	–	1108.96	–C–O–C stretching
7	1310.11	–	1301.79	–NO_2_ aromatic nitro compound
8	1398.77	1356.70	1396.99	CH_3_ deformation
9	1437.77	1401.50, 1438.70	–	In plane O–H bending and C–O stretch of dimers
10	–	1503.99, 1597.66	–	C=O stretching vibration for –COOH group
11	1603.70	–	1610.53	C=O stretching vibration for –COOH group
12	1756.77	–	1720.75	C=O stretching
13	–	2120.79	–	C–H bending
14	–	2834.91	2830.77, 2855.74	C–H stretching
15	2902.91	2933.68	–	C-H stretching
16	–	3056.44	–	O–H stretching vibration of hydroxyl functional groups
17	3323.77	3350.70	3321.66	O–H stretching vibration of hydroxyl functional groups
18	–	3756.44	–	O–H stretching vibration of hydroxyl functional groups

**Table 2.** Physicochemical characteristics of unactivated and activated biochar.

Surface Area and Pore Structure Analysis	Unactivated Char	Activated Biochar
Langmuir surface area	1.22 m^2^/g	367.10 m^2^/g
Total pore volume (Horvath-Kawazoe method)	0.0125 cc/g	0.127 cc/g
% Carbon	52.33	63.66
% Hydrogen	6.76	5.43
% Nitrogen	5.44	3.22
% Sulfur	1.39	1.03
% Oxygen	34.08	26.66

**Table 3.** The evaluation of pseudo first and pseudo second order kinetic parameters at 30°C.

Pseudo First Order Kinetics							Pseudo Second Order Kinetics				

*C*_0_ (mg/L)	*q_e_*_,_*_exp_* (mg/g)	*q_e_*_,_*_cal_* (mg/g)	*K*_1_ (min^−1^)	*h* (mg/g·min^−1^)	*R*^2^	∆*q*%	*q_e_*_, (cal)_ (mg/g)	*K*_2_ (min^−1^)	*h* (mg/g·min^−1^)	*R*^2^	∆*q*%
50	11.503	5.929	0.0184	0.109	0.886	14.6	12.048	0.0068	0.987	0.999	1.37
60	13.331	6.353	0.0184	0.117	0.833	15.8	14.048	0.0056	1.105	0.998	1.55
70	15.475	8.185	0.0230	0.188	0.811	14.2	16.393	0.0047	1.263	0.998	1.71
80	17.253	11.066	0.0276	0.305	0.877	10.8	18.182	0.0042	1.388	0.998	1.55
90	18.003	10.544	0.0230	0.243	0.977	12.7	18.519	0.0039	1.337	0.996	0.42
100	18.308	7.278	0.0161	0.117	0.898	18.2	19.231	0.0050	1.849	0.998	1.46

**Table 4.** The evaluation of the Elovich model constant at 30°C.

Initial Concentration (mg/L)	*q_e,exp_* (mg/g)	*q_e,cal_* (mg/g)	ln (*ab*)1/*b*	1/*b*	*R*^2^	∆*q*%
50	11.503	12.279	9.366	1.810	0.962	1.95
60	13.331	14.189	10.80	2.106	0.954	1.86
70	15.475	16.217	12.58	2.260	0.955	1.38
80	17.253	18.138	14.03	2.553	0.948	1.48
90	18.003	18.956	15.01	2.452	0.951	1.11
100	18.308	19.250	15.56	2.293	0.933	1.49

**Table 5.** The evaluation of the intra-particle diffusion rate constant at 30°C.

Initial Concentration (mg/L)	*q_e,exp_* (mg/g)	*q_e,cal_* (mg/g)	*C_i_*	*K_dif_* (mg/g·h^0.5^)	*R*^2^	∆*q*%
50	11.503	12.930	3.360	5.417	0.800	3.58
60	13.331	13.717	3.399	6.117	0.828	0.84
70	15.475	17.243	4.385	7.438	0.868	3.29
80	17.253	19.278	4.938	8.237	0.856	3.39
90	18.003	20.076	4.764	9.424	0.867	2.88
100	18.308	20.321	4.477	10.31	0.860	3.17

**Table 6.** Types of isotherm based on separation factor *R*_L_.

Value of *R*_L_	Magnitude	Types of Isotherm
*R*_L_ > 1	Greater than one	Unfavorable
*R*_L_ = 1	Equal to one	Linear
0 < *R*_L_ < 1	Between zero to one	Favorable
*R*_L_ = 0	Zero	Irreversible

**Table 7.** Langmuir, Freundlich and Temkin model parameters at different temperatures.

Temperature (°C)	Langmuir Isotherm				Freundlich Isotherm			Temkin Isotherm		

	*q*_max_ (mg/g)	K_L_ (L/mg)	*R*_L_	*R*^2^	*K*_F_ (mg/g)(L/mg)^1/^*^n^*	1/*n*	*R*^2^	*B*	*K*_T_ (L/mg)	*R*^2^
30	20.83	0.343	0.028	0.994	8.570	0.258	0.866	5.108	3.965	0.883
50	21.74	0.467	0.021	0.997	10.37	0.214	0.996	16.65	3.370	0.994
70	22.21	0.512	0.019	0.984	10.59	0.122	0.917	28.30	3.084	0.914

**Table 8.** Thermodynamic parameters of Cu(II) sorption onto activated char.

Temperature (K)	Thermodynamic parameters			
	∆*G*° (Kj·mol^−1^)	∆*H*° (Kj·mol^−1^)	∆*S*° (j·K^−1^·mol^−1^)	*R*^2^
303	−2.695	+8.738	+0.0202	0.923
323	−2.031	–	–	–
343	−1.911	–	–	–
